# Stem Cell Transplantation as a Dynamical System: Are Clinical Outcomes Deterministic?

**DOI:** 10.3389/fimmu.2014.00613

**Published:** 2014-12-03

**Authors:** Amir A. Toor, Jared D. Kobulnicky, Salman Salman, Catherine H. Roberts, Max Jameson-Lee, Jeremy Meier, Allison Scalora, Nihar Sheth, Vishal Koparde, Myrna Serrano, Gregory A. Buck, William B. Clark, John M. McCarty, Harold M. Chung, Masoud H. Manjili, Roy T. Sabo, Michael C. Neale

**Affiliations:** ^1^Stem Cell Transplant Program, Department of Internal Medicine, Massey Cancer Center, Virginia Commonwealth University, Richmond, VA, USA; ^2^Center for the Study of Biological Complexity, Virginia Commonwealth University, Richmond, VA, USA; ^3^Department of Microbiology and Immunology, Virginia Commonwealth University, Richmond, VA, USA; ^4^Department of Biostatistics, Virginia Commonwealth University, Richmond, VA, USA; ^5^Department of Psychiatry and Statistical Genomics, Virginia Commonwealth University, Richmond, VA, USA

**Keywords:** stem cell transplantation, dynamical system, T cell repertoire, logistic function, graft versus host disease

## Abstract

Outcomes in stem cell transplantation (SCT) are modeled using probability theory. However, the clinical course following SCT appears to demonstrate many characteristics of dynamical systems, especially when outcomes are considered in the context of immune reconstitution. Dynamical systems tend to evolve over time according to mathematically determined rules. Characteristically, the future states of the system are predicated on the states preceding them, and there is sensitivity to initial conditions. In SCT, the interaction between donor T cells and the recipient may be considered as such a system in which, graft source, conditioning, and early immunosuppression profoundly influence immune reconstitution over time. This eventually determines clinical outcomes, either the emergence of tolerance or the development of graft versus host disease. In this paper, parallels between SCT and dynamical systems are explored and a conceptual framework for developing mathematical models to understand disparate transplant outcomes is proposed.

Stem cell transplantation (SCT) represents a unique immunotherapeutic modality in which donor-derived T cells exert a graft versus host response, which when directed at host-derived malignancy, effects a cure (1, 2). However, when this phenomenon extends to normal host tissue, it results in the single most dreaded complication of this procedure, graft versus host disease (GVHD). Over the years more stringent definition of human leukocyte antigen (HLA) identity in donor–recipient pairs (DRP) has diminished the likelihood of GVHD in HLA-matched pairs undergoing unrelated donor SCT (3, 4) such that in large patient populations it is seen less frequently. But, take an individual patient – even one with a well-matched sibling donor – and it is entirely impossible to predict whether that individual will develop GVHD, requiring life-long immunosuppression, or become a tolerant chimera, able to come off immunosuppression (5, 6). Aside from the peri-transplant pharmaco-therapeutic interventions, a number of biological factors impact the risk of developing GVHD (7). These include mismatching of the minor histocompatibility antigens (mHA) (8), the cytokine milieu (9, 10), and the “regulatory” immune cell populations (11) in circulation at the time of transplantation. So despite increasing stringency of HLA matching, a substantial number of patients develop post-transplant complications, either related to GVHD or to immunosuppression (infection, relapse), contributing to therapeutic failure as evidenced by the frequent observation of high-transplant related mortality following SCT (3, 12, 13). This suggests that outcomes following SCT are inherently stochastic and subject to rules governing probability. So is there some way individual outcomes may be determined following SCT, in other words, is it possible to compute the fate of a transplant recipient?

## Do Early Conditions Affect Late Outcomes?

To ascertain this, a quantitative determination of the likelihood of various post-transplant outcomes would have to be made in different situations. As noted above, HLA matching represents a critical variable in determining survival in transplant recipients. Examining the disparity in clinical outcomes of patients transplanted using HLA matched and mismatched donors may give an indication of the quantitative effect of genetic variation at the major histocompatibility (MHC) locus and the therapeutic adjustment required to overcome that. Over the last decade, transplant outcomes observed in patients undergoing alternative donor SCT have steadily improved with relatively minor adjustments to transplant technique. As an example, poor outcomes following umbilical cord blood transplantation (UCBT) in adults were improved by infusing two cord blood units, despite the HLA mismatch between the recipients and the donor cords (14, 15). Graft loss likelihood as well as infection rates declined and no increase in GVHD was observed, even though in the long run only one of the cord blood units would engraft. In a strictly quantitative sense, if not qualitative, the stem cell dose was not significantly altered with the double cord blood infusion when compared with the dose administered using an adult donor, where it was an order of magnitude higher. Similarly, SCT from a haploidentical related donor had been consistently fraught with poor outcomes until the institution of cyclophosphamide infusion on day 3 and 4 following transplant. This has resulted in a marked improvement in survival following SCT with haploidentical donors, even in the absence of T cell depletion (16, 17). In both these examples, interventions early in the transplant course led to a lasting impact on the long-term outcome, with no further intervention beyond the norm. This occurred despite lack of HLA identity, and has led to these mismatched donor sources now being considered viable alternatives if HLA-matched donors are not available. Even when HLA-mismatched unrelated donors are considered, although the transplant risk is higher compared to an HLA-matched donor, with modern conditioning and GVHD prophylaxis regimens, survival, and GVHD incidence is relatively similar regardless of whether donors are mismatched at either the allele or antigen level (18, 19). Further, in HLA-matched unrelated donors early interventions such as infusion of anti-thymocyte globulin (20, 21) or bortezomib (22) prior to stem cell infusion have resulted in marked impact on long-term outcomes. As an example, a small difference in the dose of ATG given during conditioning may have long-term effects on the clinical endpoints occurring much later in the course of transplant, presumably by impacting immune reconstitution (23). These examples illustrate the principle that, conditions early on in the course of transplantation are critical in determining long-term outcome, to the extent that they may compensate HLA mismatch. This sensitivity to early conditions is a characteristic of deterministic systems, as opposed to systems governed by randomness.

**Figure 1:**
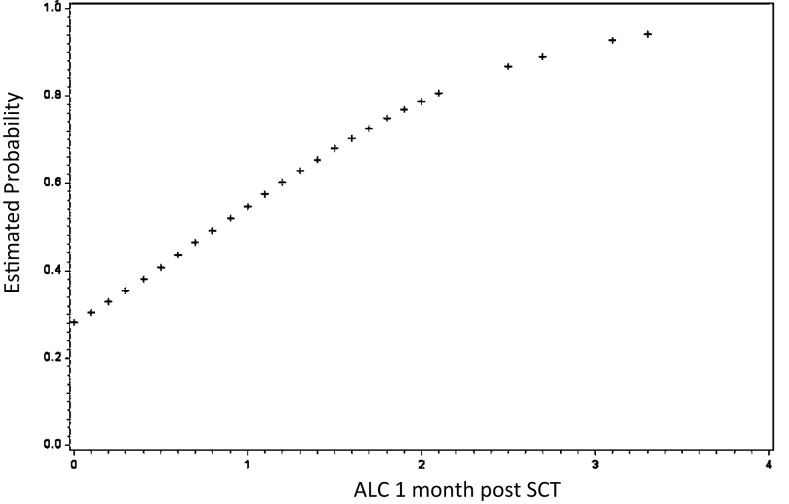
**Early lymphoid recovery influences clinical outcomes following allogeneic SCT**. Absolute lymphocyte count (ALC) at 1 month predicts survival. As 1-month ALC increased by 1/10, the odds of survival increased by over 3% (HR = 3.25; 95% CI: 1.59–6.62; *P* = 0.001). Similarly, as 1-month ALC increased by 1/10, the odds of relapse decreased by over 3% (HR = 0.33; 95% CI: 0.16–0.66; *P* = 0.002) *not shown*. Adapted from Ref. (24).

Further evidence of long-term effects of early conditions comes from examination of immune reconstitution following HLA-matched SCT. It has been a consistent observation that early donor-derived lymphoid recovery is associated with improved clinical outcomes (Figure 1); less graft loss and relapse, albeit, at the expense of greater GVHD risk (24–29). Conversely, poor donor-derived lymphoid recovery either in the form of mixed chimerism or in the terms of low-absolute lymphocyte count (ALC) puts patients at risk for eventual graft loss or relapse, particularly when reduced intensity conditioning regimens are being used (30, 31).

## Are Transplant Outcomes Deterministic?

Within patients with normal immune recovery there remains an inability to predict whether they will develop alloreactivity or not. This has been explained by the presence of different mHA outside the MHC locus. Numerous studies have documented the association of various specific mHA, or groups of mHA, with alloreactivity. However, when whole exome sequences of the SCT donors and recipients were compared, identifying *all* the single nucleotide polymorphisms in a unique DRP, and thus, the potential mHA between them, an extensive library of thousands of potential variant mHA was seen in HLA-matched pairs, making it unlikely that GVHD occurrence can be explained on the basis of *histo-incompatibility* alone (32). The large burden of minor histo-incompatibility implies that the likelihood of alloreactivity manifesting clinically may be determined by the degree of allo-antigen presentation at the time of transplant, which in turn is determined by the degree of tissue injury and inflammation. The immunosuppressive milieu at the time the initial interaction between T cells and antigen presenting cells occurs then becomes a critical factor in determining tolerance or alloreactivity emerging. The principle at hand appears to be that, all donor–recipient pairs will have immunogenic potential for alloreactivity, and in most instances very early on in the course of SCT they will be propelled on a path to certain clinical outcomes (tolerance versus GVHD versus graft loss), in a deterministic fashion.

Further support for determinism comes from immune recovery following SCT, which follows predictable kinetics in terms of the order in which various immune cell subsets reconstitute. Commonly, NK cell recovery is prompt, within a few weeks of transplantation followed by cytotoxic T cell recovery, with B cells and helper T cells lagging significantly, especially in patients undergoing T cell depletion. When T cell subsets emerging following SCT are examined with respect to the T cell receptor β (TRB) repertoire complexity, oligoclonal expansion has been observed, which over time recovers back to a more normal repertoire. Importantly, when studied using next generation sequencing (NGS), the T cell repertoire is not disordered, rather, it has a fractal ordering with respect to gene segment usage, which may be described mathematically (33). Fractals describe the geometry of many objects in nature, and are characterized by self-similarity over different scales of measurement. In the human T cell repertoire, proportionality in magnitude is maintained across scales of measurement, when T cell clonal frequency is examined in terms of TRB, variable, diversity, and joining gene segment usage. This suggests that a fractal model may be appropriate to describe immune reconstitution following SCT, strengthening the argument for SCT outcomes being deterministic. Given its immunoablative nature, SCT provides a good opportunity to examine the recovery kinetics of T cells, which appear to be influenced by the donor type and the conditions at the time of cell infusion, i.e., use of T cell depletion, or immuno-modulators. Thus, even though the rate of T cell reconstitution may vary in individuals, quantitatively it may be defined mathematically, and this implies the principle that T cell repertoire reconstitution kinetics follows a deterministic course.

## Stem Cell Transplants as Dynamical Systems

Considering these principles, sensitivity to early conditions, which in a complex background of antigenic diversity leads to different outcomes, arrived at by computable immune response; one may postulate that SCT when viewed in individual DRP is an example of a *dynamical system*. In other words, when followed over the course of time after transplantation, each future state of the transplant DRP (*system*) is dependent upon the state immediately preceding it, rather than being a random occurrence. Such dynamical systems evolve over time, and their evolution is modeled by differential equations. These systems may be precisely predictable, as in an accelerating object, where depending on the physical characteristics of the object, one would get the anticipated acceleration every time energy is applied. On the other hand, outcomes in certain dynamical systems may be more difficult to precisely predict, in other words *chaotic*, as in the case of weather, where a *complex system* influenced by a large number of variables, demonstrates disparate outcomes because its evolution over time is extremely sensitive to initial conditions. Thus, even though the behavior of chaotic systems is governed by mathematically described rules, as the system goes through successive *iterations* over time, the eventual outcomes in different individuals diverge exponentially as a function of time. This occurs because minor differences in initial conditions get magnified with the passage of time as the system evolves in each individual. The important concept to recognize in these systems is that if the initial conditions can be faithfully reproduced, chaotic systems will generally have similar outcomes each time, however, even very small fluctuations in these conditions sends the system down a different trajectory to an altogether different outcome *state* in different individuals or instances. Further, all the possible potential outcomes, or *states*, constitute the *phase space* of that system, and generally individual systems tend toward a limited number of states, mathematical entities termed “*attractors*” (34–37).

Clearly, SCT does not follow our first dynamical system model, since despite the most well designed conditioning regimens and stringently selected donors, outcomes in individual patients are highly variable. Laws of probability can give the odds of a certain outcome, but cannot chart the course an individual will follow after SCT. Further, between genomic variation between donor and recipient, donor-derived T cell repertoire, recipient cytokine milieu and microbiome as well as pathogen exposure, the number of variables to consider is much too great to expect linear, predictable behavior. Therefore, in view of the above discussion, dynamical system modeling of SCT is necessary to understand disparate outcomes, particularly when sensitivity to early conditions is borne in mind. To accomplish this differential equations describing the kinetics of T cell clonal reconstitution over time following SCT, and relating them to the eventual development of either GVHD or tolerance (relapse) may be developed to explore this idea. In such a model, the GVHD risk will depend upon the cumulative effect of the proliferating T cell clones in a deterministic fashion, rather than in a probabilistic manner.

So is it important to distinguish between stochastic or deterministic outcomes following SCT? There is an important difference between the two models: in the former, GVHD or tolerance or relapse, would develop randomly, without any underlying rule or principle being followed. In dynamical systems, however, there is an underlying set of rules that the system follows, and if the early conditions can be precisely replicated, the outcomes in different individuals will be more likely to be similar as the system evolves (Table 1). Acknowledging the difficulty of replicating initial conditions in SCT models precisely, knowledge of the rules at work in SCT would nevertheless permit measured as opposed to empiric therapeutic interventions, such as by more accurate titration and timing of cellular and pharmacotherapy to achieve desired clinical outcomes, within the limits of the system.

**Table 1:** **Characteristics of stochastic versus deterministic dynamical system modeling of SCT**.

	Stochastic	Deterministic
Determining future outcomes of SCT	Random; computed using probability function for specific outcomes determined *a priori*	Dependent on early state of the system; computed using iterating equations, such as the logistic equation
Source of variability in system outcome	Unknown extrinsic or intrinsic factors; determined by examining outcomes in large populations treated uniformly	Inherent property of growing system; mathematically determined
Parameter needs for modeling future states of the system	Limited	Extensive
Computing probability of future events	Average behavior of system in large populations	Iterating, differential equations describing behavior of system in individuals
Sensitivity to early conditions	No	Yes
Outcomes in individual with identical early conditions	Variable	Uniform
Clinical implications: GVHD likelihood at time *t_n_*_+_*_1_*	Probability function of T cell reconstitution at earlier time *t_n_*	Deterministic function of T cell reconstitution at earlier time *t_n_*
Clinical implications: post-transplant interventions	Timed according to probability of clinical outcome	Timed according to measured parameters
Clinical implications: clinical trial design	Uniform application of investigational therapy to population to determine probability distribution of resulting outcomes	Individualized therapy based on parameter variation (T cell reconstitution) and its association with desired clinical outcome

*Currently, SCT are modeled according to a limited number of known parameters such as, donor type, conditioning therapy, GVHD prophylaxis regimen, etc., in such systems, outcomes in individual transplant recipients are stochastic. In a deterministic dynamical system model, parameters such as donor–recipient genomic differences and their influence on the immune cell repertoire, and consequently on clinical outcomes are measured over time in individuals. This may allow prediction of individual clinical outcomes*.

## Evidence for the Dynamical System Model

What evidence exists that SCT represents a dynamical system? The most telling evidence is the sensitivity to early conditions; consider that in an HLA-matched SCT, minor histocompatibility antigens (mHA) are a constant presence; these are there on the first day of transplant, as they are one year later when the donor-derived T cells are fully reconstituted. Yet, bortezomib or ATG or cyclophosphamide given during conditioning may result in the development of tolerance in certain individuals, which in most instances does not break even after withdrawal of immunosuppression. Regardless of the cellular mechanism of how this is achieved, the average impact on the individual system in this instance is that the donor T cells are propelled toward a specific outcome – tolerance – which in this case would be analogous to an attractor, an endpoint to which a chaotic dynamical system tends as it evolves over time. GVHD on the other hand would represent an alternative attractor in the system. An example of this is seen in lymphoid (T cell and NK cell) recovery during the first 2 months following SCT, influencing eventual outcomes following SCT, whether they be survival, relapse or GVHD (Figure 1) (14, 15, 38). The system “trajectory” or output may be modified by an intervention; such as donor lymphocyte infusion (DLI) or intensification of immunosuppression to treat GVHD, but left to itself it will tend toward one of the “attractors.”

**Figure 2:**
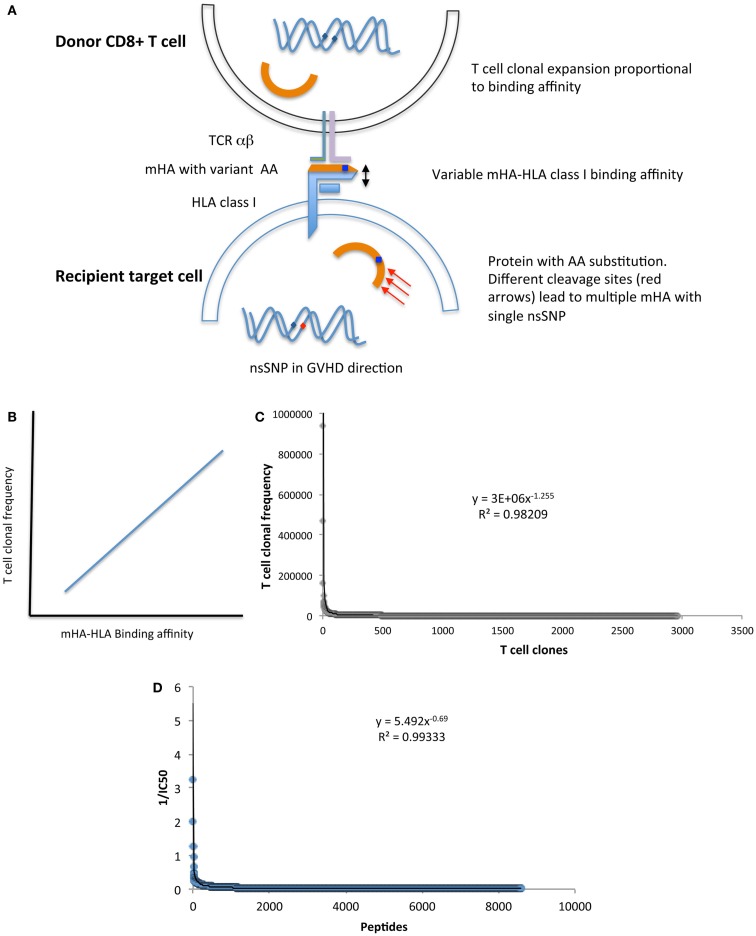
**(A)** Model depicting the relationship between donor T cell clonal frequency and recipient mHA-HLA binding affinity. **(B)** Postulated association between peptide-HLA binding affinity and T cell clonal frequency distribution. **(C)** T cell clonal frequency distribution^1^ and **(D)** the values of reciprocal of IC50^2^ (mHA-HLA binding affinity estimate) for mHA-HLA in a single DRP. Both parameters follow a Power law distribution, suggesting that peptide-HLA affinity spectrum has an important role in determining T cell repertoire. Footnotes:^1^ T cell clonal frequency measured on day 100 post SCT, by high throughput sequencing of T cell receptor β, cDNA obtained from CD3^+^ cells, given in copy number of unique clones and arranged in descending order with a cutoff at <100 copies. ^2^1/IC_50_ of mHA-HLA complexes-(estimate of the binding affinity), determined by whole exome sequencing to identify nsSNPs between donor and recipient in the GVH direction, followed by *in silico* determination of the resulting oligopeptide sequence, and the IC50 of the resulting mHA-HLA complexes.

**Figure 3:**
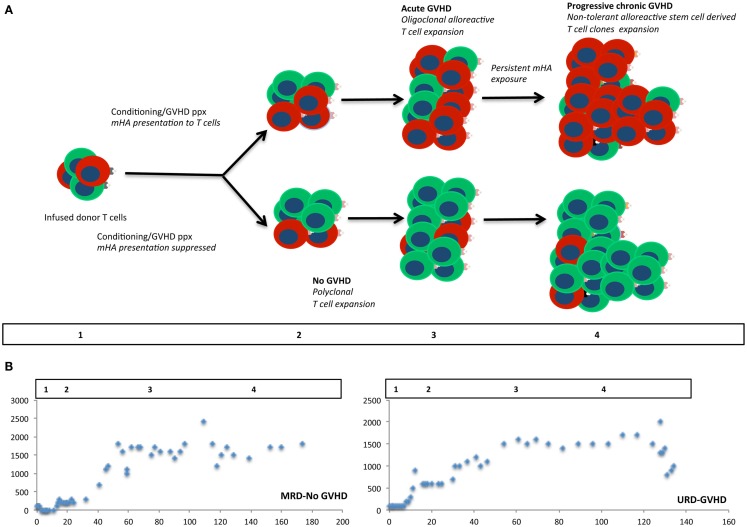
**Modeling stem cell transplantation as a dynamical system**. Iterative expansion of donor T cells clones over time in the presence of an alloreactivity potential, modulated by the degree of antigen presentation. In **(A)** cells colored red, represent alloreactive T cell clones, and green cells, other non-alloreactive T cell clones. Allo-antigen exposure or lack thereof in the first few days of transplant results in minor early differences in the repopulating T cell clones, which over time results in an exponential expansion of corresponding T cell clones. Different phases of cellular proliferation are labeled 1, 2, 3, and 4 in the schema and extrapolated to the plots depicting absolute lymphocyte counts from two patients following SCT **(B)**. These plots show a bi-logistic growth pattern, reflecting initial engraftment and cessation of mycophenolate mofetil following SCT.

Additional support for a chaotic model of SCT comes from the fractal organization of T cell repertoire. Chaotic systems may be represented geometrically as fractals, which demonstrate iterating patterns across scales of magnitude. T cell clonal frequency when considered in terms of TRB, variable, joining, and diversity gene segment usage has a fractal organization. This results in a complex repertoire comprised of thousands of T cell clones, which when examined in terms of clonal frequency, follow a Power distribution, characteristic of self-similarity across scales of measurement, at all levels of TRB clonal definition (33). Further, when the genomic variability between donors and recipients is considered (32), and translated into putative mHA, the binding affinity of the resulting peptides to the relevant HLA demonstrates a non-linear, Power law distribution, reminiscent of the T cell clonal distribution (Figure 2) (39). Therefore, one may postulate that the driving force behind T cell reconstitution after SCT is the spectrum of binding affinities of recipient mHA (and pathogen) peptides with the relevant HLA in the individual transplant DRP, encountered by donor T cells in the recipient. This is possibly the case, as is evident in a comparison of the peptide-HLA binding affinity distribution and T cell clonal frequency distribution from two different studies performed by our group (Figures 2C,D). In this model, depending on the initial conditions following SCT (T cell dose infused + cytokine milieu + pharmacotherapy) specific donor T cell clones will proliferate or decline in a deterministic manner. The antigen presentation in the very beginning will result in either alloreactive or pathogen specific T cell clones proliferating over time, and will eventually determine the clinical outcome, either tolerance or GVHD (Figure 3).

## Modeling T Cell Clonal Expansion in SCT

One model that may describe the cellular immune recovery following SCT is the logistic model of growth first described by Verhulst in 1838 to explain population dynamics. Logistic growth is described by an equation of the form:

$$x_{t+1}=r\;x_{t}\left(1-x_{t}\right).$$

In this equation, population size (*N*) at discrete intervals of time (*t*, *t* + *1*, *t* + *2* …) is represented as a ratio, *x*, of the possible maximum population size at a much later time *t_n_* (carrying capacity, *K*). This ratio (*x* = *N/K*), at any given time in the evolution of a population (for example, *x_t_*_+_*_1_*) is always determined by the population ratio from an earlier time (*x_t_*). In this *iterating equation* the term, *r* represents the maximum intrinsic growth rate of the population and is called the “*driving parameter*” (36, 40). This relationship has several implications; first, as the population (in this case clonal frequency of individual T cell clones) grows over time, its size at some final time will depend on both the size of the starting population at *t*_0_, and the value of *r*. Second, after an initial period of exponential growth, the growth rate slows down asymptotically because the term (1 −* x_t_*) becomes smaller as the population increases. Third, as the value of *r* increases, the variance observed in *x* over time increases, eventually behaving in a chaotic manner. This is depicted in the Logistic Map, where the values *x* takes on in the long-term, are plotted against *r* (http://mathworld.wolfram.com/LogisticMap.html). This demonstrates that while the value of *x* diminishes to zero over time when *r* is <1, a steady increase in the value of *x* is observed as *r* goes from 1 to 3; at *r* > 3 and <3.5, the system may take on two different sets of values of *x* (bifurcation), consistent with a population oscillating between two extremes; and finally, at *r* > 3.5 the system behaves chaotically with large and unpredictable variation in the value of *x* (and *N*) over time. Despite this seemingly chaotic behavior, however, if the logistic map is examined at ever-smaller scales (higher decimal place values of *r*) the bifurcation patterns of *x* seen in the larger map are reproduced in a self-similar manner at each scale of magnification, revealing hidden structure in the distribution of *x* with each increment in *r*, in other words, fractal organization. If individual T cell clones are considered as unique populations, this provides a plausible explanation for the fractal T cell repertoire observed in SCT recipients.

Extrapolating this model to individual T cell clones followed over time after SCT, one would observe very different growth rates depending on the parameter *r* governing the growth of each clone. And even though the proliferation of the T cell clones follows deterministic rules, chaotic behavior (if *r* is high enough) means that, though the eventual clonal frequency of unique T cell clones will be difficult to predict precisely, the overall repertoire will demonstrate underlying order, as was observed in the fractal ordering of the T cell repertoire. Further, the independence of *x* from *N* in the logistic equation means that as the Logistic function iterates for each clone, relative proportionality is maintained between T cell clonal populations as they vary over time, resulting in the scale invariance characteristic of fractal geometry. In such a model, the individual T cell clones may differ in their frequency by orders of magnitude (41), however, this can be addressed by employing a more complete and complex model of growth, such as the Gompertz curve, which by taking *Log x*, accounts for the logarithmic nature of growth in biological systems. A potential additional advantage of this is that, it may describe sigmoid population growth more accurately than the Logistic growth curves while also explaining chaotic growth behavior (42, 43).

**Figure 4:**
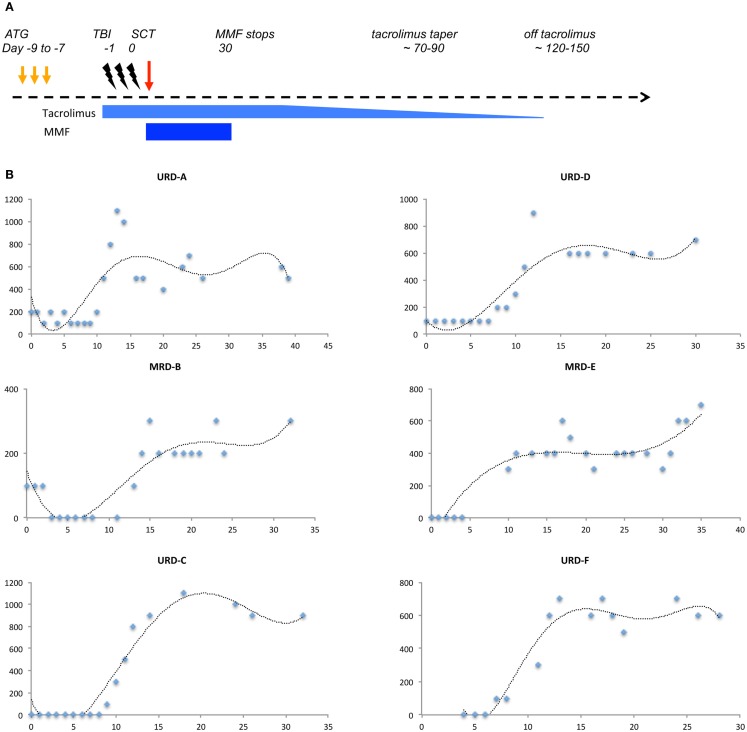

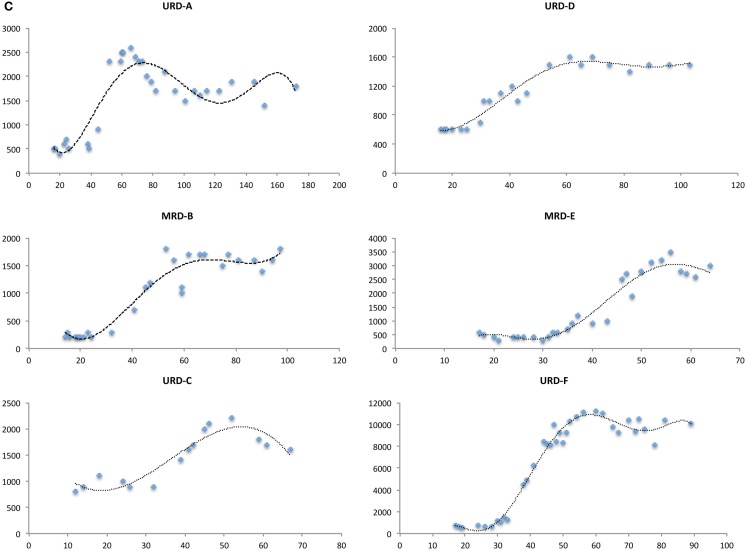

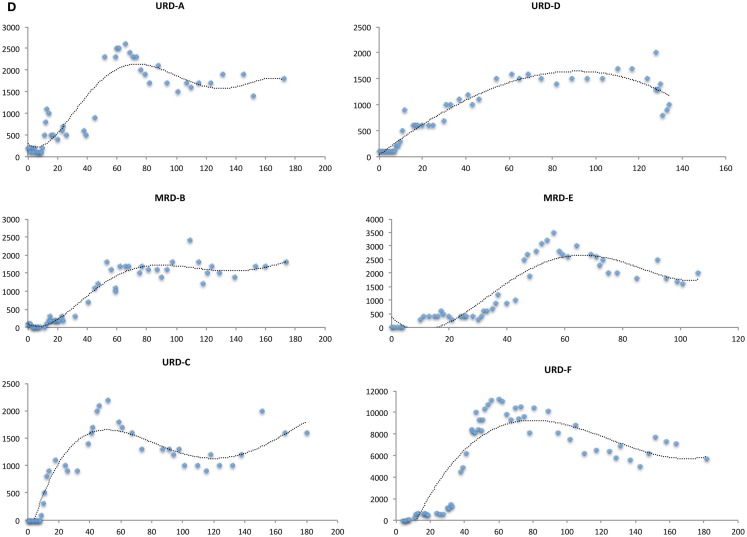

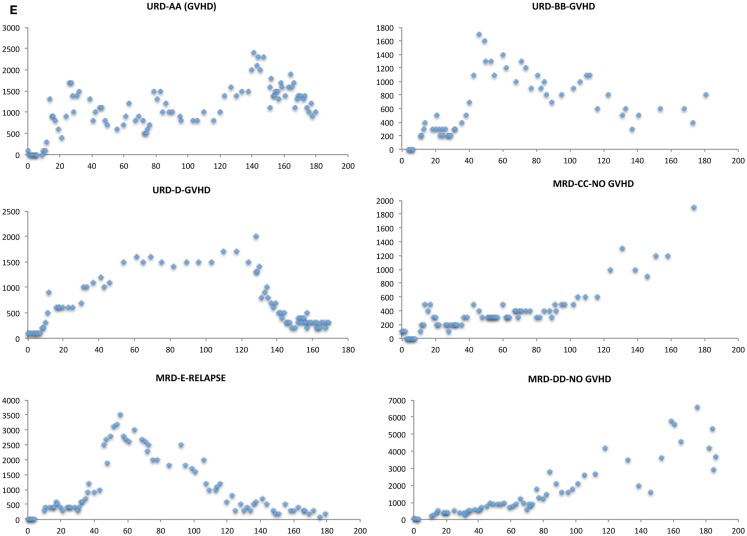
**Absolute lymphocyte count (ALC, μL^−1^) plotted as a function of time (days following transplant)**. **(A)** Schema of the transplant protocol, outlining the general immunosuppression withdrawal scheme. **(B)** ALC in the first month following SCT shows the first growth phase coincident with engraftment. **(C)** ALC in the second and third month following SCT shows the second exponential growth phase following cessation of MMF, of these patients only patient D developed GVHD. **(D)** ALC in the first 4–6 month following SCT shows the overall growth kinetics of lymphocytes. Data in all these plots may be modeled with a logistic equation of the general form, *N_t_* = *K/(1* + *Ae*^−^*^rt^)*, where *A* = *(K − N_0_)/N_0_*, where *N*_0_ represents the lymphocyte count at the beginning, and *Nt* is the lymphocyte count at time *t* following transplant, *e* is the base of natural logarithms, 2.718 and *r* is the growth rate of the population. A similar equation, *N_t_* = *N_0_* + *(K − N_0_)/(1* + *10^(a−t)r^)*, where *a*, is the time at which growth rate is maximal and an inflection point is observed in the logistic curve, also describes the data. **(E)** Patients with clinical events, depicting impact of immunosuppressive therapy on lymphocyte counts, and departure from the sigmoid growth patterns. Also, seen is the variability from measurement to measurement in ALC in the fourth month, when comparing patients with GVHD (AA and D) and those without (CC and DD).

This hypothesis is supported by the lymphocyte reconstitution kinetics observed in patients undergoing MRD and URD SCT using an immunoablative reduced intensity conditioning (Clinicaltrial.gov identifier: NCT00709592). In this regimen, thymoglobulin (either 5 or 7.5 mg/kg) was administered in divided doses from day −9 to −7 followed by 4.5 Gray fractionated total body irradiation. GVHD prophylaxis was with tacrolimus (day −2 to approximately day 120) and mycophenolate mofetil (MMF; day 0–30) (Figure 4A). In patients who achieved hematopoietic engraftment, lymphocyte reconstitution could be plotted as a function of time using a sigmoidal growth curve described by the logistic equation. Most patients had two discernable periods of exponential increase in ALCs, one following engraftment (Figure 4B), and another period following cessation of MMF (Figure 4C). These growth periods were followed by a plateau with relatively stable lymphocyte counts in the absence of clinical events, until withdrawal of tacrolimus when greater variability was observed (Figure 4D). Patients developing complications of therapy such as relapse, viral (CMV or EBV) reactivation, and GVHD requiring immunosuppression, such as corticosteroid therapy, had significant departures from the sigmoidal curve, as did patients with delayed engraftment kinetics (Figure 4E). It should be recognized that this data represents *all* the lymphocyte subsets, such as NK, T, and B cells. The initial phase is likely largely derived from NK cell recovery while the secondary phase most likely represents T cell reconstitution. The latter postulate is consistent with the finding that the day 60 *donor-derived CD3*+ cell count is predictive of clinical outcomes (25). It is logical that total lymphocyte counts would reflect the kinetics of T cell clonal proliferation, and that this should be governed by the same principle that describes population dynamics in general, i.e., it is a logistic function of time. Therefore, lymphocyte count growth rate (*r*) observed in our cohort may represent an average of the growth rate of thousands of T cell clones following SCT. Thus, a simple logistic growth model of T cell reconstitution may be developed which would explain GVHD occurrence as an exponential increase in alloreactive T cells, when immune reconstitution is considered as an iterative process over time (Figure 3).

In SCT, *r* for each T cell clone may depend upon multiple variables, including the antigen-HLA specificity of the T cell receptor (Figure 2A), the immunosuppressive regimen being used and the cytokine milieu during the period of growth as well as the proportion of regulatory T cell clones. Further, it may vary as immunosuppression is withdrawn following SCT (Figure 4A) or inflammatory states, such as CMV reactivation or GVHD develop leading to increasingly chaotic behavior of the T cell clones. On the other hand, rate of change of *x* will depend not only on the infused T cells, but will vary as hematopoietic precursors engraft and depending on thymic integrity differentiate into immune cell populations. It is important to recognize that in this dynamical immune reconstitution model the chaotic behavior is occurring at the level of the *individual T cell clones*, and while individual clones may demonstrate marked variance in their frequency over time following transplant, it is their cumulative effect, which, results in GVHD or tolerance. If a large number of mHA directed T cell clones proliferate, then the consequence would be GVHD. Conversely, if non-mHA directed T cell clones dominate, tolerance ensues with immune reconstitution (Figure 3). In such a hypothetical system, the total T cell count trends reflect the average effects of this phenomenon, and the clinical outcomes are an effect of this chaotic expansion of individual T cell clones, with GVHD and tolerance serving as the attractors. It may be postulated that the restoration of a more “complete” fractal structure will result in optimal clinical outcome. Studies demonstrating oligoclonal T cell expansion in patients with GVHD or relapse demonstrate the validity of this hypothesis (44–47). This concept is testable by measuring the fractal dimension of the post-transplant T cell repertoire by high-throughput sequencing (39). Therefore, if one can account for the complexity at hand in SCT, perhaps by using NGS to study the antigenic variance between donors and recipients, as well as T cell clonotypes following SCT, it will likely be well described as a chaotic dynamical system. Serial high-throughput sequencing of TRB may allow plotting of the T cell clonal frequency as it evolves over time following SCT, resulting in a plot which would yield a fractal surface expanding over time, as individual T cell clones vary and new clones emerge. Such analyses will likely be valuable in distinguishing different prognostic groups of patients on the basis of post-transplant T cell repertoire reconstitution.

Using the dynamical system model, one might monitor the rate of change, in other words *r*, for the dominant T cell clones following SCT and correlate this with clinical manifestations to determine if this is associated with outcomes. Accurate mathematical modeling of the dynamical evolution of T cell repertoire following SCT would allow for *measured*, and *earlier* therapeutic intervention in the event of either GVHD or inadequate immune recovery or residual disease. For example, this may be an intervention using more intense or prolonged post-transplant immunosuppression, for patients with rapid rate of change of *x* or those with a high value of *r*, to reduce the risk of GVHD by reducing the chaotic behavior if a large number of T cell clones have a high *r*. Conversely, DLI may be similarly used when the opposite conditions prevail. Further, knowledge of the critical time periods when exponential T cell clonal growth occurs with different transplant regimens will allow optimal timing of interventions such as vaccination. For instance, vaccination may be best given before the exponential rise in T cell numbers begins in order to maximize utilization of cytokines, and minimize potential competition between T cell clones. This concept is utilized in lympho-depleting chemotherapy regimens used as a part of adoptive immunotherapy (48). Therefore, considering SCT as a dynamical system rather than a stochastic one, would allow logic based patient management and quantitative trial designs minimizing empiric interventions. As such, therapy may be designed for individual patients based on a systematic and personalized approach, instead of relying on population-based outcomes derived from probabilistic study designs. In essence, the development of accurate mathematical models that account for the key variables influencing transplant outcomes has the potential to improve clinical outcomes following SCT, making SCT an even better example of personalized medicine than it already is.

## Conflict of Interest Statement

Dr. Amir Ahmed Toor has received research support from Sanofi-Aventis manufacturers of Thymoglobulin. The other co-authors declare that the research was conducted in the absence of any commercial or financial relationships that could be construed as a potential conflict of interest.

## Supplementary Material

The Supplementary Material for this article can be found online at http://www.frontiersin.org/Journal/10.3389/fimmu.2014.00613/abstract
