# Supplementary material for: Stem Cell Transplantation as a Dynamical System: Are Clinical Outcomes Deterministic?
Source: Front Immunol. 2014 Dec 3;5:613. doi: 10.3389/fimmu.2014.00613 (PMC4253954; doi:10.3389/fimmu.2014.00613)
Supplement: Supplementary file 1 [file Data_Sheet_1.PDF]

Appendix I

Modeling stem cell transplantation as a dynamical system. An analogy with a system based in physics will help illustrate this point more clearly, where streams of elementary particles and their secondary emissions are influenced by the electromagnetic fields that they travel in, through time. Transplantation with donor-derived T cells, responding over time to alloreactivity potential (recipient immunogenic mHA-HLA) under the influence of conditioning and immunosuppressive therapy may be similarly considered. Considering a cathode ray tube apparatus, the stream of electrons (e) symbolizes *donor T cells*, the electrical field (E) deflecting the electrons, depicts the *alloreactivity potential* (a function of the immunogenic recipient peptide-HLA complexes that donor T cells encounter), the magnetic field (H) represents the *conditioning and immunosuppressive therapy* that a patient undergoes. The Target is analogous to *tissues* initially encountered by donor T cells, with secondarily emitted electrons (e') illustrating the *donor T cell proliferation*, upon encountering *tissue specific alloreactivity potential* (E') under the influence of diminished *immunosuppression* (H') later in the course of transplantation. Finally, the Signal denotes the *clinical outcome* observed, and like the signal intensity versus location may be normally distributed, with graft loss and fatal GVHD representing the extremes and range of tolerance and milder forms of GVHD, making up the middle.

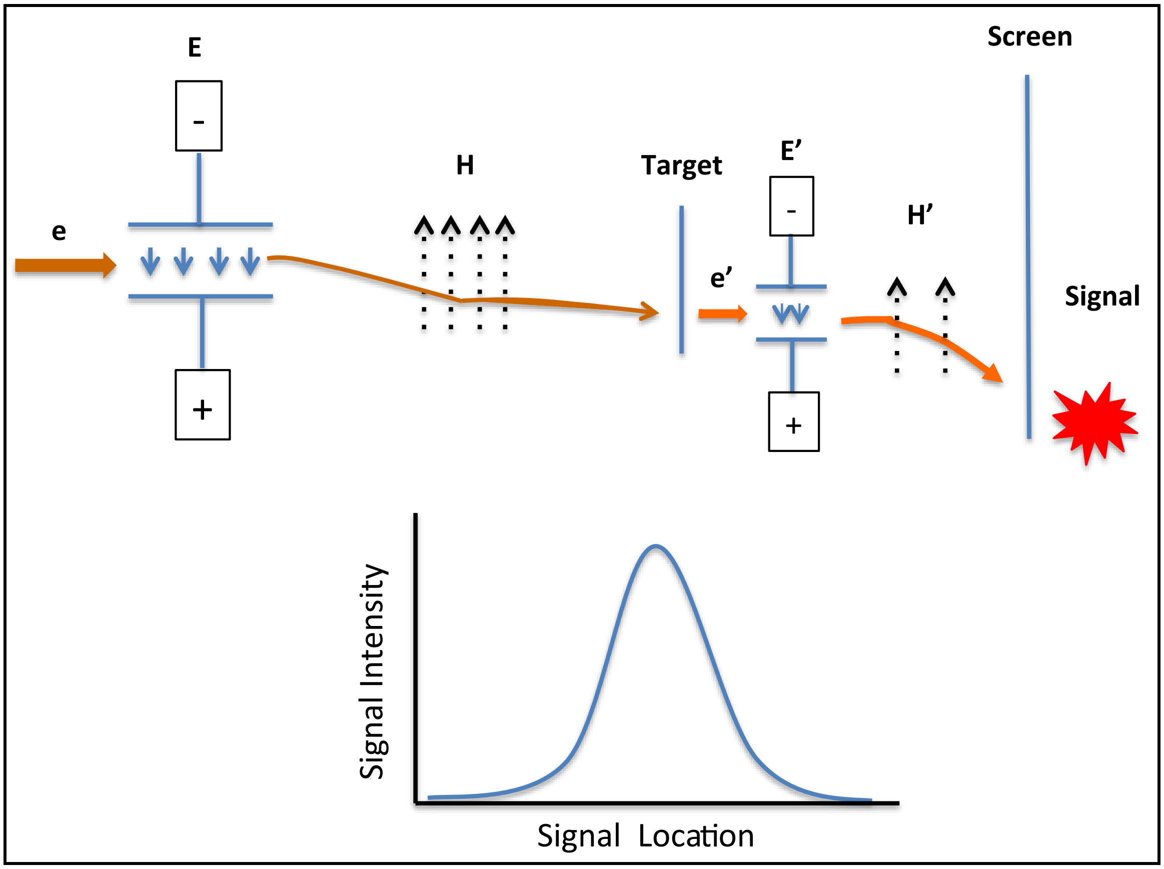

Appendix II

Modeling stochastic versus deterministic evolution of the T cell repertoire. Consider the T cell repertoire evolution as an iterative process occurring after SCT, and measured at successive times, $t_0 \rightarrow t_1 \rightarrow \rightarrow t_n$, depicted in a series of matrices. For this model, it may be assumed that GVHD is a function of the cumulative effect of the alloreactive T cell clones (*marked by *)* present at a high frequency at any given time. In a *stochastic model*, the T cell clonal frequency, measured by TCR-β V (*2, 3, 7 & n*) and J (*x, y, z & m*) segment sequencing, will evolve randomly and be determined at each time by the probability function which will be unique for each clone at each time point, for instance, P_0, P_1, P_2 and P_n. Alloreactive T cell clonal proliferation, would occur randomly with a certain probability distribution, making GVHD incidence a probabilistic outcome. On the other hand, in a *deterministic model*, the T cell clonal frequency at each time will depend on the clonal frequency at the preceding times, and be governed by the initial conditions and the growth parameters of the logistic equation. The growth rate (*r*) for each clone will be unique depending on factors such as the target antigen-HLA binding affinity, cytokine milieu and overall state of immunosuppression at the given times. Early on after transplant the conditions may be such that a large number of alloreactive T cell clones start proliferating, and become dominant following exponential growth. GVHD occurrence, being a function of the cumulative frequency and number of alloreactive clones, will then be highly likely (*GVHD attractor*). Some patients may yet escape GVHD because as the *r* for each T cell clone goes up, the different T cell clones start proliferating in a chaotic manner, with resulting large fluctuations in the clonal frequencies and GVHD may fail to develop if the alloreactive clonal populations happen to decline in unison (*tolerance attractor*).

T cell repertoire at SCT, t_0

	Vβ 2	Vβ 3	Vβ 7	Vβ n
Jβ.1.1	$x2(t_0)^*$	$x3(t_0)$	$x7(t_0)$	$xn(t_0)$
Jβ 1.2	$y2(t_0)$	$y3(t_0)^*$	$y7(t_0)$	$yn(t_0)$
Jβ 1.3	$z2(t_0)$	$z3(t_0)$	$z7(t_0)^*$	$zn(t_0)$
Jβ n	$m2(t_0)$	$m3(t_0)$	$m7(t_0)$	$mn(t_0)^*$

T cell repertoire following SCT, t_1

	Vβ 2	Vβ 3	Vβ 7	Vβ n
Jβ.1.1	$x2(t_1)^*$	$x3(t_1)$	$x7(t_1)$	$xn(t_1)$
Jβ 1.2	$y2(t_1)$	$y3(t_1)^*$	$y7(t_1)$	$yn(t_1)$
Jβ 1.3	$z2(t_1)$	$z3(t_1)$	$z7(t_1)^*$	$zn(t_1)$
Jβ n	$m2(t_1)$	$m3(t_1)$	$m7(t_1)$	$mn(t_1)^*$

T cell repertoire following SCT, t_n

	Vβ 2	Vβ 3	Vβ 7	Vβ n
Jβ 1.1	$x2(t_n)^*$	$x3(t_n)$	$x7(t_n)$	$xn(t_n)$
Jβ 1.2	$y2(t_n)$	$y3(t_n)^*$	$y7(t_n)$	$yn(t_n)$
Jβ 1.3	$z2(t_n)$	$z3(t_n)$	$z7(t_n)^*$	$zn(t_n)$
Jβ n	$m2(t_n)$	$m3(t_n)$	$m7(t_n)$	$mn(t_n)^*$

As depicted in Figure 2, the 1/IC50 and the T cell clonal frequency have a Power Law distribution, it may be therefore be logically inferred that the driving parameters (*r*) for all the logistic equations for the T cell clones above, when plotted will have a Power Law distribution as well. This means that in the evolving T cell repertoire after SCT, a number of T cell clones will

have very high value of r, and will display chaotic behavior. A large number of T cell clones will have mid-range values of r, and will follow standard growth kinetics, achieving a steady state after a phase of exponential expansion. Finally a large number of donor T cell clones may have a low driving parameter value resulting in those clones diminishing over time in terms of frequency. Assuming that GVHD development is a function of the number, frequency and activation status of allo-reactive T cells, the chaotically behaving T cells may then be the most important determinant of the apparently 'random' occurrence of GVHD. This may be measured by examining T cell clonal frequency variation over time by serial determination of TCR β high-throughput sequencing after transplantation. Clinically this means that aside from setting the early conditions such that alloreactive T cell clones will be diminished to induce tolerance, ongoing immunosuppression will be critical to suppress the chaotic behavior of T cell clonal populations after transplantation and reduce the likelihood of GVHD onset.
